# Effects of Comprehensive Bundled Nursing Care on Psychological Disorders in Mechanically Ventilated Intensive Care Unit Patients

**DOI:** 10.62641/aep.v54i4.2242

**Published:** 2026-08-15

**Authors:** Yanyan Zhang, Yun Chen

**Affiliations:** ^1^Department of ICU, The First People’s Hospital of Linping District, 311100 Hangzhou, Zhejiang, China

**Keywords:** patient care bundles, intensive care units, mechanical ventilation, anxiety, depression, cognitive dysfunction

## Abstract

**Background:**

This study aimed to explore the effects of comprehensive bundled nursing care on psychological disorders (anxiety, depression, post-traumatic stress disorder [PTSD], and cognitive impairment) in mechanically ventilated patients in the intensive care unit (ICU). In addition, the study analysed the effect of these nursing strategies on patient treatment adherence, providing evidence for optimizing psychological nursing strategies in the ICU.

**Methods:**

This retrospective study included 116 ICU patients who received mechanical ventilation. They were divided into two groups based on the type of nursing care: the bundled comprehensive nursing group (n = 58) and the control group (n = 58). The control group received routine nursing care, whereas the bundled comprehensive nursing group received comprehensive nursing care, which included psychological care as the core component, supplemented by position optimization, oral care, and nutritional support. Psychological and cognitive outcomes were assessed using the Hamilton Anxiety Rating Scale (HAMA), Hamilton Depression Rating Scale (HAMD), Post-traumatic Stress Disorder Checklist (PCL-C), and Montreal Cognitive Assessment (MoCA) before and after nursing care. Additionally, treatment adherence was compared between the two groups.

**Results:**

Before the nursing care, no significant differences were found between the two groups in any of the above scales (all *p* > 0.05). After the nursing care, the bundled comprehensive nursing group showed significantly lower HAMA, HAMD, and PCL-C scores and higher MoCA scores compared with the control group (all *p* < 0.001). In addition, treatment adherence was significantly improved in the intervention group (*p* < 0.01).

**Conclusions:**

The bundled comprehensive nursing was associated with reduced anxiety, depression, and PTSD symptoms, improved cognitive function, and enhanced treatment adherence in mechanically ventilated ICU patients. This study provides an important clinical reference for optimizing psychological nursing strategies in the ICU.

## Introduction

Mechanical ventilation is a critical clinical intervention for critically ill patients. It provides respiratory support to those with respiratory distress, improves respiratory quality, and reduces mortality risk [1, 2]. However, due to their severe condition, invasive procedures and specialized environment, intensive care unit (ICU) patients on mechanical ventilation are exposed to multiple stresses—including discomfort and separation from family making them highly susceptible to psychological disorders [3, 4]. A recent systematic review showed that the pooled prevalence of post-traumatic stress disorder (PTSD) among ICU survivors was 19.83%, while the prevalence of anxiety and depression reached 32%; mechanical ventilation is an independent risk factor for these psychological disorders [5]. Dyspnea in the ICU is often closely associated with anxiety. It not only increases patients’ psychological burden but also prolongs the duration of mechanical ventilation, affects long-term quality of life, and raises the risk of PTSD after hospital discharge. These patients commonly exhibited remarkable anxiety, which frequently co-occurred with depression, PTSD, and cognitive decline [6].

These psychological issues not only reduce treatment adherence but may also delay recovery, increase healthcare burdens, and substantially impair patient prognosis [7]. Therefore, exploring targeted nursing models to ameliorate the psychological state of mechanically ventilated ICU patients has become a major challenge in critical nursing care. Bundled comprehensive nursing is an evidence-based nursing model that provides comprehensive, systematic care by integrating a series of targeted nursing measures [8]. Its core advantage lies in balancing physiological comfort with psychological support, enabling personalized care plans tailored to individual patient conditions to enhance nursing outcomes [9]. A systematic review by Mastrogianni *et al*. [10] Reported that nursing bundles have been widely used in adult ICUs to prevent ventilator-associated pneumonia (VAP), with core interventions including semi-recumbent positioning, oral hygiene, subglottic suctioning, and cuff pressure control. The review included 38 studies and showed that standardized nursing bundles reduced the incidence of VAP by more than 36%, with some bundles achieving near-zero VAP rates, indicating high effectiveness and clinical value of the nursing bundle.

Based on this, the current study employs bundled comprehensive care in mechanically ventilated ICU patients, focusing on its effects in improving anxiety, depression, PTSD, and cognitive impairment. This study aims to provide practical evidence for optimizing ICU psychological care strategies and enhancing patients’ mental health levels.

## Methods

This retrospective cohort study was reported in accordance with the Strengthening the Reporting of Observational Studies in Epidemiology (STROBE) guideline to ensure transparency, standardization, and completeness of the study design, methods, and results (**Supplementary Table 1**). This study was performed in line with the principles of the Declaration of Helsinki. Ethical approval was granted by the Ethics Committee of the First People’s Hospital of Linping District, Hangzhou (Approval No. 069, 2023). Written informed consent was obtained from patients or their legal representatives, depending on the patient’s clinical condition at the time of enrolment.

### Clinical Data

This retrospective analysis was conducted on medical records of 116 mechanically ventilated patients admitted to the ICU of the First People’s Hospital of Linping District, Hangzhou between December 2020 and March 2021.

Based on the nursing care documented in the medical records, patients who received bundled comprehensive nursing were assigned to the bundled comprehensive nursing group (n = 58), whereas those who received routine clinical nursing were assigned to the control group (n = 58). Group classification was determined according to actual clinical nursing records, without additional artificial grouping or nursing care.

### Inclusion and Exclusion Criteria

Inclusion criteria: (1) patients who met the indications for mechanical ventilation; (2) Acute Physiology and Chronic Health Evaluation II (APACHE II) scores ≥10 points [11]; (3) Patients with complete clinical data; (4) patients underwent mechanical ventilation for the first time with a duration of ≥48 h [12]; and (5) Conscious patients, i.e., those with a Glasgow Coma Scale (GCS) score ≥ 13, indicating that patients were conscious and able to cooperate with assessment [13].

Exclusion Criteria: (1) Patients with a history of chest malignancy and chest trauma; (2) Patients with severe and uncontrollable hemodynamic instability requiring continuous vasoactive support; (3) Patients with severe and uncontrollable pulmonary infection or moderate to severe acute respiratory distress syndrome (ARDS); (4) Patients with hematologic diseases or autoimmune diseases; or (5) Patients with spinal cord injury.

### Nursing Methods

Patients in the control group received routine nursing care. The indwelling tubes and catheters were adequately protected and properly fixed. The temperature and humidity in the ward were adjusted. If a patient underwent airway incision, the two layers of sterile gauze would be covered over the tracheostomy cannula, and the humidification solution would be dripped as directed by the doctor. The patient’s wound would be regularly disinfected, and the gauze would be replaced regularly. Sputum suction was performed, life care was strengthened, and daily living assistance was provided to the patients, and patients’ vital signs were closely monitored.

Based on the control group, the bundled comprehensive nursing group implemented bundled comprehensive nursing as follows:

(1) Postural nursing. Body position was adjusted according to the patient’s condition. If there was no special contraindication, the head of the patients’ bed was raised appropriately by 30°–45°.

(2) Respiratory nursing. The patients’ respiratory circuits were regularly replaced once a week, and the condensed water was timely disposed of. The condensed water accumulation bottle was maintained below the level of the ventilator’s circuit. The liquid in the humidification tank and atomizer was regularly treated once a day, and terminal disinfection was carried out at the same time. Patients’ vital signs were closely monitored, and sputum suction nursing care was provided according to the specific situation. The duration of sputum suction should be less than 15 seconds. The subglottic secretions were sustainedly aspirated, and then volume, quality, and colour of the secretions were routinely assessed. The air bag pressure was maintained at 20–25 cmH_2_O, and the air bag needed to be relaxed every 6 hours.

(3) Oral nursing patients’ mouths were rinsed and scrubbed. The rinse solution was taken and injected slowly from the corner of the mouth on one side of the patient, and the opposite side of the mouth was simultaneously sucked with negative pressure.

(4) Aseptic operation and infection prevention. The principle of aseptic operation was strictly followed. The seven-step hand washing method was strictly implemented before operation. Ultraviolet disinfection was performed daily. Beds and instruments were wiped and disinfected with 500 mg/L chlorine-containing disinfectant, three times a day. The air was cultured weekly, and the number of bacteria in the air should be less than 200 CFU/m^3^.

(5) Nutritional support. A personalized diet plan was formulated according to the patient’s own condition, and enteral nutrition combined with parenteral nutrition support was provided.

(6) Psychological nursing. The nursing care was implemented by full-time psychological nurses holding professional qualifications and with at least 5 years of experience in ICU psychological care. The nursing care was conducted once daily for 20–30 minutes each session and was adjusted according to the patient’s tolerance. The specific procedures consisted of four components: (a) cognitive nursing care, including explanations of the purpose, precautions, and successful cases of mechanical ventilation to reduce negative perceptions; (b) emotional support, alleviating anxiety and fear through verbal comfort and non-verbal communication (e.g., touch, eye contact); (c) relaxation training, including guided breathing exercises and muscle relaxation to reduce stress responses; and (d) family support, including 1–2 video calls with family members daily to enhance emotional support. The entire process was documented in detail to ensure standardization and reproducibility.

### Observation Indicators

All assessments were completed by two trained independent researchers using observer-rated scales rather than patient self-reports to address potential issues such as sedation, delirium, and communication difficulties in ICU patients. Standardized time points were uniformly adopted in the study: baseline assessments were conducted within 2 hours after enrolment, and post-care assessments were conducted within 2 hours before weaning or discharge, which effectively reduced measurement bias.

The reliability of all scales was confirmed in the clinical populations. Cronbach’s α coefficients were as follows: Hamilton Anxiety Rating Scale (HAMA), 0.86; Hamilton Depression Rating Scale (HAMD), 0.88; Post-traumatic Stress Disorder Checklist (PCL-C), 0.90; Montreal Cognitive Assessment (MoCA), 0.85; and Morisky Medication Adherence Scale-8 (MMAS-8), 0.82. All coefficients were above 0.80, indicating good internal consistency and reliability of these scales in the ICU population. All scale scores were derived from archived assessment records within the medical record system and were not conducted additionally for this study.

#### Primary Outcomes

(1) Anxiety Level: The HAMA [14, 15] was used to assess the anxiety status of patients in both groups before and after nursing care. This scale consists of 14 items, with a total score ranging from 0 to 56 points, where higher scores indicate more severe anxiety. (2) Depression level: The Hamilton Depression Rating Scale 17-item version (HAMD-17) was used to assess the depression level, with a total score ranging from 0 to 52 points. A higher score indicated more severe depression [16]. (3) PTSD: The PCL-C [17] scale consists of 17 items, with a total score range of 17–85 points. Each item is scored on a 1–5 scale. The assessment timing is synchronized with HAMD, allowing the trauma-related psychological status to be recorded simultaneously. (4) Cognitive Function: The MoCA [18] was used to evaluate the effect of nursing care on cognitive function amelioration. It covers multiple cognitive domains, including attention and concentration, executive functions, memory, language, visual construction ability, conceptual thinking, calculations, and orientation, with a total score range of 0–30 points. A score ≥26 indicates normal cognitive function, while a score <26 indicates cognitive impairment. Higher scores reflect better cognitive function. MoCA scores were recorded once before and after nursing care.

#### Secondary Outcome

Assessment was performed using the MMAS-8 [19] combined with records of adherence with nursing procedures. The MMAS-8 consists of eight items with total scores ranging from 0 to 8. A score of ≥8 points indicates full adherence, 6–7 points indicates partial adherence, and a score of ≤5 points indicates non-adherence. Concurrently, a comprehensive evaluation was conducted based on nursing care records extracted from medical charts, including the completion status of key procedures such as body position management, oral care, and respiratory circuit maintenance. The overall adherence rate was calculated using the formula: Overall Adherence Rate = (Number of full adherence + Number of partial adherence) / Total number of patients × 100%.

### Bias Control

This was a retrospective study based on clinical nursing records. Therefore, the following measures were taken to control selection bias and information bias:

(1) Patients were assigned to groups according to the actual nursing method they received during hospitalization, without prospective random grouping, artificial allocation, or additional intervention. (2) Uniform and strict inclusion and exclusion criteria were applied to select cases, ensuring the baseline data of the two groups were as balanced and comparable as possible, thereby reducing selection bias. (3) All data were independently and retrospectively extracted from medical records by two researchers, and a third researcher reviewed any discrepancies to ensure the accuracy and completeness of the data. (4) All scale assessments were derived from routine clinical evaluation records rather than being conducted specifically for this study, and the assessment process followed unified clinical standards, which reduced measurement bias.

### Statistical Approaches

Data analysis was performed by SPSS 21.0 (IBM Corp., Armonk, NY, USA). The Shapiro–Wilk test was used to assess the normality of continuous variables. Categorical variables were reported as frequencies and percentages [n (%)], and pairwise comparisons were conducted using the Chi-square (χ^2^) test; Normally distributed continuous variables were expressed as mean ± standard deviation ($\bar{x}$ ± s) and compared between groups using the independent-samples *t*-test, and within-group comparisons were performed using the paired-samples *t*-test. Non-normally distributed data were expressed as median (interquartile range) and analysed using the Mann–Whitney U test. A two-sided test was adopted, and *p* < 0.05 was considered statistically significant.

## Results

### Baseline Characteristics of the Two Groups

In the bundled comprehensive nursing group, there were 19 female patients and 39 male patients with an average age of (60.12 ± 6.88) years; the APACHE II scores were (16.12 ± 2.28) points. The length of ICU stay was (7.35 ± 2.16) days, and the duration of mechanical ventilation was (73.26 ± 12.53) hours. There were 21 female and 37 male patients in the control group, with an average age of (60.59 ± 6.69), and the APACHE II scores were (16.77 ± 2.01) points, the length of ICU stay was (7.51 ± 2.23) days, and the duration of mechanical ventilation was (74.15 ± 13.08) hours. After statistical software testing, the baseline characteristics of the two patient groups did not differ significantly (*p* > 0.05) (Table 1).

**Table 1.** **Analysis of the baseline data of the two groups**.

Group	n	Gender: (male/female)	Age (years)	APACHE II scores	Length of ICU stay (days)	Mechanical ventilation time (h)
Bundled comprehensive nursing group	58	39/19	60.12 ± 6.88	16.12 ± 2.28	7.35 ± 2.16	73.26 ± 12.53
Control group	58	37/21	60.59 ± 6.69	16.77 ± 2.01	7.51 ± 2.23	74.15 ± 13.08
χ^2^ / *t*		0.153	0.465	1.653	0.582	0.419
*p*		0.696	0.643	0.101	0.561	0.676

ICU, intensive care unit; APACHE II, Acute Physiology and Chronic Health Evaluation II.

### Comparison of HAMA Scores Before and After Nursing Care Between the Two Groups

As shown in Table 2, before nursing care, the HAMA score in the bundled comprehensive nursing group was 18.56 ± 3.32, while that in the control group was 18.43 ± 3.26, with no significant difference between the two groups (*p* > 0.05). After nursing care, the HAMA score in the bundled comprehensive nursing group decreased significantly to 9.12 ± 2.15 compared with the levels before nursing care (*p* < 0.001). Similarly, the control group exhibited a significant reduction in HAMA score to 11.35 ± 2.28 after nursing care (*p* < 0.001). Moreover, after nursing care, the HAMA score in the bundled comprehensive nursing group was significantly lower than that in the control group, with the difference reaching statistical significance (*p* < 0.001).

**Table 2.** **Comparison of Hamilton Anxiety Rating Scale (HAMA) scores before and after nursing care between the two groups**.

Group	Sample size	Before nursing care	After nursing care	*t*/F value	*p* value	*t*/F value (inter-group)	*p* value (inter-group)
Bundled comprehensivenursing group	58	18.56 ± 3.32	9.12 ± 2.15	22.897	<0.001	6.234	<0.001
Control group	58	18.43 ± 3.26	11.35 ± 2.28	15.678	<0.001	-	-

### Comparison of Scores on Multiple Psychological Scales Before and After Nursing Care

As shown in Table 3, before nursing care, comparisons of HAMD, PCL-C, and MoCA scores between the two groups revealed no significant differences (*p* > 0.05), indicating comparability. After nursing care, both groups showed significant reductions in HAMD and PCL-C scores and significant increases in MoCA scores compared with baseline (all *p* < 0.001). Specifically, the HAMD score (8.76 ± 2.34) and PCL-C score (32.12 ± 4.56) were significantly lower than those in the control group (11.56 ± 2.45 and 38.76 ± 4.89, respectively), with significant intergroup differences (*t* = 6.234, 7.123; both *p* < 0.001). Concurrently, the MoCA score (27.89 ± 2.15) in the bundled comprehensive nursing group was significantly higher than that in the control group (24.56 ± 2.34), with significant differences between groups (*t* = 8.765, *p* < 0.001).

**Table 3.** **Comparison of scores on multiple psychological scales before and after nursing care**.

Scale	Group	Before nursing care ($\bar{x}$ ± s)	After nursing care ($\bar{x}$ ± s)	Intra-group *t*-value	Intra-group *p*-value	*t*/F value between groups	*p*-value between groups
HAMD	bundled comprehensivenursing group (n = 58)	19.23 ± 3.56	8.76 ± 2.34	23.012	<0.001	6.305	<0.001
	Control group (n = 58)	18.98 ± 3.45	11.56 ± 2.45	16.025	<0.001		
PCL-C	bundled comprehensivenursing group (n = 58)	45.67 ± 5.89	32.12 ± 4.56	16.987	<0.001	7.123	<0.001
	Control group (n = 58)	44.98 ± 5.76	38.76 ± 4.89	8.976	<0.001		
MoCA	bundled comprehensivenursing group (n = 58)	22.34 ± 2.67	27.89 ± 2.15	12.345	<0.001	8.765	<0.001
	Control group (n = 58)	22.12 ± 2.56	24.56 ± 2.34	6.543	<0.001		

HAMD, Hamilton Depression Rating Scale; PCL-C, Post-traumatic Stress Disorder Checklist; MoCA, Montreal Cognitive Assessment.

### Nursing Adherence

The overall adherence rate in the bundled comprehensive nursing group was 96.55%, with 34 full adherence, 22 partial adherence, and 2 non-adherence. In the control group, 21 patients were classified as full adherence, non-adherence, 25 as partial adherence, and 12 were non-adherence, resulting in an overall adherence rate of 82.76%. The overall nursing adherence rate in the bundled comprehensive nursing group was significantly higher than in the control group (*p* < 0.05) (Table 4).

**Table 4.** **Contrast of nursing adherence between the two groups [n (%)]**.

Group	n	Full adherence	Partial adherence	Non-adherence	Total adherence
Bundled comprehensive nursing group	58	34 (58.62)	22 (37.93)	2 (3.45)	56 (96.55)
Control group	58	21 (36.21)	25 (43.10)	12 (20.69)	46 (79.31)
χ^2^					8.942
*p*					0.003

## Discussion

Currently, mechanical ventilation is widely applied in the clinical management of critically ill patients, providing effective respiratory support for those with respiratory distress, improving respiratory quality, and laying the foundation for disease treatment and recovery [20, 21]. As an evidence-based nursing approach, bundled comprehensive care is characterized by proactivity, specificity, scientific rigor, and purposefulness. Its core advantage lies in integrating multidimensional nursing care that addresses both patients’ physiological and psychological needs, thereby enhancing care quality through systematic approaches.

In the present study, the bundled comprehensive nursing group, building upon routine care, implemented physiological nursing care, including postural care, respiratory care, oral care, aseptic techniques, and nutritional support. These measures may have enhanced patients’ physical comfort, reduced the accumulation of negative emotions, thereby creating more favourable conditions for the implementation of psychological nursing care.

The bundled comprehensive nursing group also demonstrated significantly higher treatment adherence compared with the control group (96.55% vs. 82.76%). This outcome is closely linked to the systematic nature of bundled care. Physiological comfort alleviated patients’ physical distress, while targeted psychological nursing care ameliorated their mental state. The combined effect of these approaches may have contributed to enhance patients’ cooperation with treatment, ultimately providing crucial support for better overall care outcomes [22].

It should be noted that the mean APACHE II score of the patients included in this study was approximately 16 points, indicating a moderate-to-severe illness severity, with a significantly increased risk of in-hospital death. Despite this, the bundled comprehensive nursing group achieved a high treatment adherence rate (96.55%). These results may be attributed to the comprehensive and standardized design of the bundled comprehensive nursing protocol, which improves patients’ physical comfort and psychological status. This finding is consistent with previous clinical studies showing that standardized ICU bundled nursing care helps improve treatment adherence in critically ill patients [23].

The physiological care measures in the bundled comprehensive nursing group serve as adjunctive means to improve patients’ physical comfort and reduce the psychological burden caused by discomfort, thereby establishing a stable physiological foundation for the core psychological nursing care and better helping patients alleviate negative emotions. In addition, the standardized oral care in this study has been confirmed by multiple high-quality studies [24, 25] to improve oral health status and reduce the risk of ventilator-associated complications. A multicentre, stepped-wedge, cluster randomized controlled trial (the CHORAL study) by Dale *et al*. [26] showed that discontinuing chlorhexidine and implementing a standardized oral care bundle could effectively improve oral health and reduce infection-related ventilator-associated complications in patients receiving mechanical ventilation, thereby decreasing physical stressors and indirectly alleviating negative emotions such as anxiety and fear, providing a more stable physiological basis for the implementation of psychological nursing care.

After the nursing care, HAMA scores in both groups were significantly lower than before the nursing care (*p* < 0.001), suggesting that both nursing methods could alleviate patient anxiety to some extent. Notably, the after-nursing care HAMA score in the bundled comprehensive nursing group was 9.12 ± 2.15, which was significantly lower than that in the control group (11.35 ± 2.28). These results indicate that bundled comprehensive nursing based on routine nursing can more effectively reduce the anxiety level of ICU patients undergoing mechanical ventilation. Multidimensional cluster nursing built on psychological assessment, ward environmental regulation and continuous emotional communication can effectively mitigate patients’ overall negative psychological reactions, which aligns with the general conclusion from Di *et al*. [27] that standardized cluster nursing interventions relieve diverse adverse psychological symptoms in critically ill ICU populations.

Meanwhile, a recent clinical trial also confirmed that a structured comprehensive nursing intervention, including elevated head of bed, standardized oral care, subglottic secretion drainage, and cuff pressure management, significantly reduced the incidence of VAP among mechanically ventilated patients in the ICU, with a relative risk reduction of up to 67% [28]. Standardized bundled comprehensive nursing reduces adverse stressors such as infection, airway irritation, and physical discomfort, creating a more stable physiological state for patients, thereby indirectly alleviating negative emotions such as anxiety and fear. These findings are highly consistent with the psychological improvement results of the current study.

Beyond anxiety assessment, the present study further evaluated multidimensional psychological assessments of depression, PTSD, and cognitive function. After the nursing care, the HAMD and PCL-C scores in the bundled comprehensive nursing group were significantly lower than those in the control group, and the MoCA score was significantly higher than that in the control group. These results suggest that the bundled comprehensive nursing may be associated with broader psychological benefits beyond anxiety reduction alone. The observed improvements may be related to the integrated psychological nursing care throughout the entire process. The comprehensive multi-scale assessment within 24 hours of admission enables accurate identification of various psychological problems and formulation of individualized plans. Daily professional psychological counselling and cognitive behavioural therapy directly correct negative cognitions, and relaxation training helps patients actively regulate stress responses. Video communication with family members and optimization of the ward environment provide emotional support, reduce loneliness and discomfort, and indirectly improve post-traumatic psychological status and cognitive function. In contrast, routine nursing lacks a systematic psychological nursing care framework, has limited effects on improving psychological status, and cannot meet patients’ multidimensional psychological needs. Bundled comprehensive nursing organically combines physiological comfort measures with psychological support nursing care, while ensuring a safe basic treatment environment, precisely targets the multiple psychological distresses caused by the disease and treatment, and ultimately achieves comprehensive optimization of psychological status, providing a more complete practical approach for psychological nursing of ICU patients [29].

This study still has certain limitations. First, this was a single-centre retrospective study with a relatively small sample size, which may limit the generalizability of the findings and introduce selection bias. Future multicentre, large-sample, prospective studies are needed to further validate the generalizability of the conclusions. Second, this study did not include long-term follow-up, so it cannot assess the long-term effects of nursing care on patients’ psychological status and quality of life after discharge. Subsequent studies should extend the follow-up duration for long-term prognostic observation. Third, this study did not perform subgroup analyses based on ages, disease severity, or ventilation duration, nor did it explore differences in individualized nursing care. Future stratified or individualized nursing studies could be conducted to develop more precise psychological nursing protocols for ICU patients undergoing mechanical ventilation. Fourth, this study compared multiple psychological indicators (HAMA, HAMD, PCL-C, MoCA) between groups without correction for multiple comparisons, which may increase the probability of type I error. The results should be interpreted with caution, and future studies could use correction methods (e.g., Bonferroni) to further improve the robustness of statistical findings. Finally, this study did not collect or adjust for potential confounding factors such as medication use, sedation and analgesia, or psychotropic medications, and thus cannot completely exclude their influence on patients’ anxiety, depression, cognition, and other outcomes. The effectiveness of bundled comprehensive nursing still needs to be further validated through studies that control for additional confounding factors.

## Conclusions

This retrospective study suggests that a comprehensive bundled nursing care was associated with reduced symptoms of anxiety, depression, and PTSD, as well as improved cognitive function and treatment adherence in mechanically ventilated ICU patients. These benefits support the clinical value of integrating physiological comfort measures and targeted psychological support into routine ICU practice. However, the findings should be interpreted in light of the study’s limitations, including its single-centre retrospective design, potential selection bias and residual confounding factors. Future large-sample, multicentre, prospective randomized controlled studies with long-term follow-up are necessary to validate these findings and provide a stronger evidence base for the psychological care of mechanically ventilated critically ill patients.

## Availability of Data and Materials

The datasets used and/or analysed during the current study were available from the corresponding author on reasonable request.

## Supplementary Material

Supplementary material associated with this article can be found, in the online version, at https://doi.org/10.62641/aep.v54i4.2242.
