# Supplementary material for: Effects of Comprehensive Bundled Nursing Care on Psychological Disorders in Mechanically Ventilated Intensive Care Unit Patients
Source: Actas Esp Psiquiatr. 2026 Aug 15;54(4):1159–67. doi: 10.62641/aep.v54i4.2242 (PMC13571203; doi:10.62641/aep.v54i4.2242)
Supplement: Supplementary file 1 [file ActEsp-54-4-1159-1167-s1.zip › Supplementary Table 1.docx]

# Appendix S1 Observational Study Reporting Standards followed in this study

| **entry** | **English Translation** | **Corresponding location of this study** | **Completion**  **status** |
| --- | --- | --- | --- |
| 1 | The title and abstract clearly state the study design | Title, Abstract | Completed |
| 2 | Elaborate on the research background, rationale, and scientific questions | First paragraph of Introduction | Completed |
| 3 | Clearly define the research objectives and hypotheses | End of Introduction | Completed |
| 4 | Specify the type of study design | 1 Data and Methods | Completed |
| 5 | Specify the study setting and time frame | 1.1 Clinical Data | Completed |
| 6 | Clearly define the study participants, inclusion, and exclusion criteria | 1.2 Inclusion and Exclusion Criteria | Completed |
| 7 | Clearly define the study participants, inclusion, and exclusion criteria | 1.1 Clinical Data | Completed |
| 8 | Define the study variables and measurement methods | 1.4 Observation indicators | Completed |
| 9 | Specify the data sources and assessment tools | 1.4 Observation indicators | Completed |
| 10 | Describe in detail the  interventions/exposure factors | 1.3 Nursing methods | Completed |
| 11 | Clearly define the outcome measures  and assessment time points | 1.4 Observation indicators | Completed |
| 12 | Specify the methods for controlling confounding factors and bias | 1.5 Bias control | Completed |
| 13 | Describe the statistical analysis methods and software used | 1.6 Statistical approaches | Completed |
| 14 | Describe the participant enrollment and exclusion process | 1.1 Clinical data | Completed |
| 15 | Report the baseline characteristics of the study participants | Table 1: Baseline data | Completed |
| 16 | Report the primary outcome data and effect estimates | 4Tables 2, 3, 4 | Completed |
| 17 | Report the results of between-group comparisons and statistical tests | 2 Results section | Completed |
| 18 | Describe the subgroup / supplementary analyses performed | 3 Discussion | Completed |
| 19 | Discuss the limitations of the study | End of 3 Discussion | Completed |
| 20 | Interpret the findings in the context of existing evidence | 3 Discussion | Completed |
| 21 | Explain the generalizability and clinical significance of the study | 3 Discussion | Completed |
| 22 | Declare the funding sources and conflicts of interest | End of the text | Completed |
